# Interval time between neoadjuvant chemotherapy and surgery in advanced gastric cancer doesn't affect outcome: A meta analysis

**DOI:** 10.3389/fsurg.2022.1047456

**Published:** 2023-01-16

**Authors:** Yuhao Zhai, Zhi Zheng, Wei Deng, Jie Yin, Zhigang Bai, Xiaoye Liu, Jun Zhang, Zhongtao Zhang

**Affiliations:** Department of General Surgery, Beijing Friendship Hospital, Capital Medical University, Beijing, China

**Keywords:** neoadjuvant chemotherapy, advanced gastric cancer, interval time, pathological complete response, surgery

## Abstract

**Background:**

The efficacy of neoadjuvant chemotherapy for advanced gastric cancer is not yet firmly confirmed, but the exciting results demonstrated in several clinical studies have led neoadjuvant chemotherapy as the important treatment methods in guidelines. The 4–6 weeks interval time is currently the most commonly used in clinical treatment, but there are insufficient studies to support this time and the optimal interval has not yet been identified. The aim of this meta-analysis was to investigate the short-term life quality and long-term prognostic impact of the interval time between the end of neoadjuvant chemotherapy and surgery in patients with advanced gastric cancer.

**Methods:**

We conducted a systematic literature search in PUBMED, Embase and Cochrane Liabrary for studies published or reported in English from January 2006 to May 2022. We summarised relevant studies for the time to surgery (TTS), included as retrospective studies and prospective studies. The primary study outcome was the rate of pathological complete response (pCR), and the secondary outcomes included R0 resection rate, incidence of serious postoperative complications, 3-year progression free survival time (PFS) rate and overall survival time (OS) rate. TTS were classified in three groups: 4–6 weeks, <4 weeks and >6 weeks. The ratio ratios (ORs) were calculated and forest plots and funnel plots were made to analysis by using fixed-effect and random-effect models in Review Manager 5.2.

**Results:**

A total of five studies included 1,171 patients: 411 patients in shorter TTS group (<4 weeks), 507 patients in medium TTS group (4–6 weeks) and 253 patients in longer TTS groups (>6 weeks). And The results of our meta-analysis indicate that there are no significant difference between the three groups. The pCR, R0 resection rate, incidence of serious postoperative complications, 3-year PFS and OS were similar between three groups.

**Conclusions:**

Although there many studies exploring the suitable TTS in advanced gastric cancer, but we have not find the evidence to prove the TTS is the risk factor influencing the outcome.

**Systematic Review Registration:**

https://www.crd.york.ac.uk/PROSPERO/, identifier: CRD42022369009

## Introduction

Gastric cancer (GC) is one of the most common cancers in the world, and it is the third leading cause of death among all cancers in China (1). The symptoms of GC are not obvious and lack specificity in early stages; thus, most patients are already in the advanced stage at the time of initial diagnosis. Surgery is still the most important treatment and the first choice for those advanced gastric cancer patients currently, but it is not recommended to get operation immediately if the tumor is more difficult to be radical resected by surgery and the surgery may do more harms than the help to the patients. Neoadjuvant chemotherapy emphasises the combination of the pre-operative chemotherapy and surgery, with the aim of reducing tumour size, achieving tumour downstaging, providing the surgery possibility for neoadjuvant chemotherapy patients and reducing the difficulty of surgery.

The concept of neoadjuvant chemotherapy was firstly introduced by Frei et al. (2) in 1982 and subsequently applied in the treatment of solid tumours, such as lung cancer and esophageal cancer. The neoadjuvant chemotherapy for gastric cancer is becoming an important treatment modality after the MAGIC study (3).

The NCCN guidelines for gastric cancer recommend neoadjuvant chemotherapy for patients with locally advanced gastric cancer (T3–4, N+, M0) without distant metastases, and then the radical resection surgery and lymph node dissection should be carried following the neoadjuvant chemotherapy to ensure the treatment effects (4).

For neoadjuvant chemotherapy in gastrointestinal diseases, TTS is often considered as a factor that reflects the probability of pathological complete response to chemotherapy (5–7). In rectal cancer, the extended interval between neoadjuvant treatment and surgery increased the rate of pathological complete response. In the study of esophageal cancer, if giving a long TTS (>9 weeks), it showed the better pathological response rates and DFS compared to the shorter group, but there is still many drawbacks to this study: there was no significant difference between the two groups in terms of OS (8).

There are no clear criteria for TTS in gastric cancer and most large scale clinical studies have been performed at 4–6 weeks TTS considering the physical condition and pCR rate. Yi Liu et al. conducted a retrospective study of 176 patients and divided them into <4 weeks, 4–6 weeks and >6 weeks groups according to the interval time. It was found that the 4–6 week group was statistically different from the >6 week group in terms of PCR, but did not show a difference in 3-year survival (9). After that many studies that include postoperative recovery and complication rate were carried, and postoperative survival were the concerns of these studies (10). However, none of them have a unified view to guide clinical work and the efficacy of TTS for GC patients still needs to be further explored and clarified. In this view, whether the interval for neoadjuvant chemotherapy needs to be strictly limited needs to be explored, and whether the current most common used: 4–6 week interval regimen is optimal still needs to be discussed.

Due to these controversies, the aim of our meta-analysis was to assess the impact of TTS on the prognosis of advanced gastric cancer and also to explore whether the 4–6 weeks interval which were currently commonly used in clinical practice is superior compared to other time periods.

## Materials and methods

### Search strategy and selection criteria

Before May 1, 2022, We searched the databases Pubmed and the Cochrane Central Register of Controlled Trials and used key index terms: (gastric or gastric cancer or gastric carcinoma or cancer of stomach) and (preoperative chemotherapy or Neoadjuvant chemotherapy) and (resection or surgery or operation or gastrectomy) and (interval or timing or time or elapse or delay).

**Figure 1:**
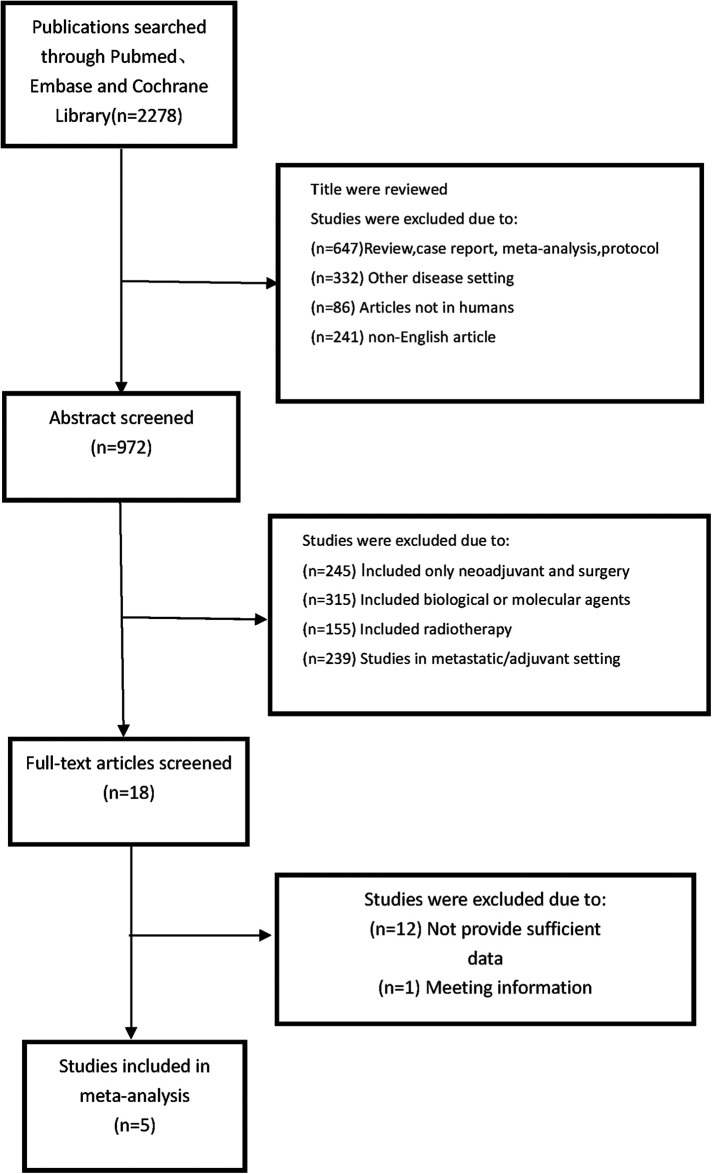
PRISMA 2009 flow diagram of the selection of included studies.

The eligibility criteria followeds., based on the PICOS strategy (Population, Intervention, Comparison, Outcome, Study): (1) the population consisted of patients who underwent NACT followed by surgery (2) Comparing differences in efficacy and outcome between shorter different TTS groups (3) the outcomes that including overall survival (OS) and pCR rate or other endpoints. (4) retrospective or prospective cohort studies. (5) original articles published in English between January 2006 and May 2022. We included the studies as these steps: (1) Publications searched through Pubmed, Embase and Cochrane Library; (2) Abstract screened and selection; (3) Screening the full text and assessing; (4) making the final decision. The detailed steps of our search for studies are shown in Figure 1.

### Data extraction

The Newcastle–Ottawa scale were used to assess all the included studies. This scale assessed the quality of studies on a scale of 0–9 and ≥6 score studies were regarded as the high quality study.

The data were extracted by two independent investigators from all eligible studies. If there were Controversies, the problem should be solved by the third investigator with communication. And we collected the following data from those studies: Two investigators independently extracted data from all eligible 5 studies. Controversial problems were resolved by discussion with another investigator.

The following data were collected from each study: pathologic complete response (pCR) rates, and if available, R0 resection rates, 3-year PFS, 3-year OS, severe postoperative mortality rates (Clavien-Dindoda ≥3). The author's name and the year of the study also should be recorded. EngaugeDigitizer 4.1 (http://digitizer.sourceforge.net/) were used to access the survival rates (PFS and OS) if there were only Kaplan–Meier survival curves provided.

### Statistical analysis

The patients were divided into 3 groups according to the TTS between NACT and surgery. The primary endpoint was the pCR rates and secondary endpoints included OS, PFS, the rates of R0 resection and severe postoperative mortality rates. We chose the odd ratios (ORs) as the principal summary measures and 95% confidence intervals (CI) also should be shown in this study. If the *P*-values <0.05 in two sides, we considered that the difference were statistically significant. We carried out the analysis and choose the fixed-effect/Mantel–Haenszel model or random-effect/DerSimonian–Laird model according the heterogeneity results which were estimated by Q-test and I2 test (Q-test *P* < 0.05 or *I*^2^ > 50%). The meta analysis was completed by using the Review Manage 5.2 software and Stata 13.0 software for bias test. The forest plot and protocols were prepared by RvMan to provide more information about those studies.

## Result

Among the 2,278 publications searched by the criteria, a total of 5 studies (9–13) met all the eligibility requirements and were included. And in Table 1, we showed these studies' baseline information and Newcastle–Ottawa scale. The studies were selected from several countries, including China, Lithuania and Spain. According to the Newcastle Ottawa scale, all eligible studies' quality were high, that 3 had 7 score and 2 had 8 score, that met our requirements. All eligible studies were retrospective studies and were published between 2006 and 2022. There are 1,171 patients included in the studies, with 411 patients in shorter TTS group (<4 weeks), 507 patients in medium TTS group (4–6 weeks) and 253 patients in longer TTS groups (>6 weeks). The baseline information between the each groups were no significant difference, and all the studies included the patients with advanced gastric cancer (stage II–IV). The tumor location and other information which may influence the efficacy of neoadjuvant chemotherapy were similar between the groups. We made the funnel plots that showed the symmetry around the axis of the treatment effect for the end-points, which indicated that there was no publication bias.

**Table 1:** Characteristics of the included studies.

Study	Year	Country	Study design	Sample	Cutline of interval	Male (%)	Age (median or mean)	Stage	Quality score
Augustinas Bausys	2021	Lithuania	Retrospective	280	ESG:<30 days (70) SSG:31–43 days (138) DSG: >44 days (72)	ESG:39 (55.7) SSG:79 (57.2) DSG:44 (61.1)	ESG:62 SSG:64 DSG:62	II–IV	7
Chaorui Wu	2019	China	Retrospective	229	≤4 weeks (70) 5–6 weeks (103) >6 weeks (56)	≤4 weeks 49 (70.0) 5–6 weeks 75 (72.8) >6 weeks 33 (58.9)	≤4 weeks 58 5–6 weeks 55 >6 weeks 57	II–IV	8
Juan Ocaña	2020	Spain	Retrospective	60	<4 weeks (18) 4–6 weeks (26) >6 weeks (16)	<4 weeks 7 (38.9) 4–6 weeks 16 (61.5) >6 weeks 9 (56.3)	<4 weeks 65.56 4–6 weeks 65.69 >6 weeks 66.75	II–IV	8
Yi Liu	2017	China	Retrospective	176	<4 weeks (111) 4–6 weeks (48) >6 weeks (17)	<4 weeks 87 (78.38) 4–6 weeks 37 (77.08) >6 weeks 13 (76.47)	<4 weeks 55.6 4–6 weeks 59.8 >6 weeks 59.8	II–IV	7
Yinkui Wang	2020	China	Retrospective	426	≤21 days (49) 22–28 days (93) 29–35 days (108) 36–42 days (84) 43–84 days (92)	≤21 days 36 (73.47) 22–28 days 71 (76.34) 29–35 days 82 (75.93) 36–42 days 65 (77.38) 43–84 days 73 (79.35)	≤21 days 61 22–28 days 60 29–35 days 59.5 36–42 days 61.5 43–84 days 63	II–IV	8

ESG, the early-surgery group; SSG, the standard-surgery group; DSG, the delayed-surgery group.

### pCR rates

**Figure 2:**
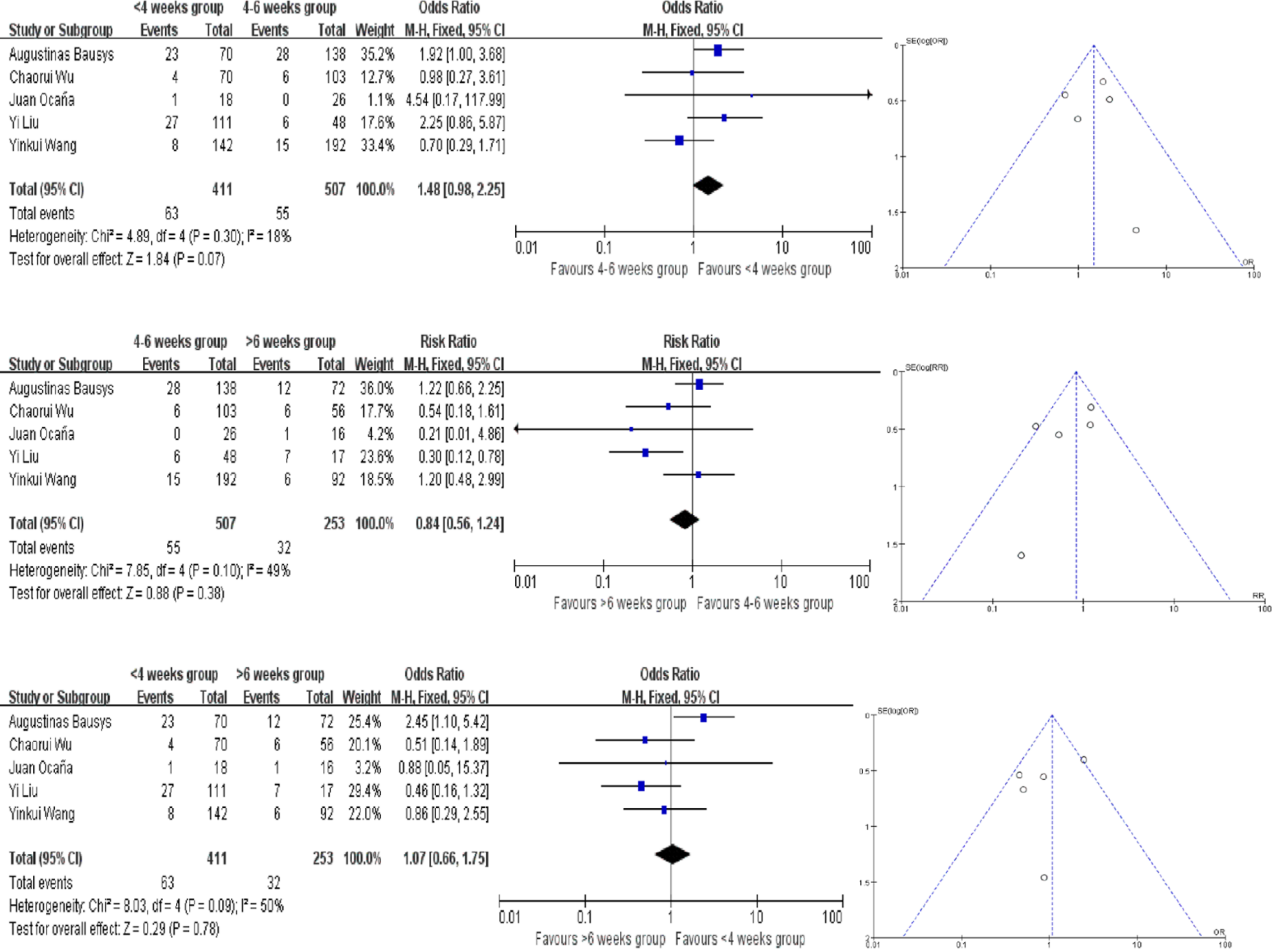
Forest plot and funnel plot for pathologic complete response (pCR) rates meta analysis.

4 of the studies provided the pCR rate, while Augustinas Bausys defined a new definition: mPR in his study, which may be regarded as another kind of pCR based on the Becker tumor response system. Our meta analysis showed no difference in pCR rates in there groups. Compared the <4 weeks and 4–6 weeks groups, the heterogeneity testing was moderate with *I*^2 ^= 18% and the *P* for heterogeneity = 0.30 using the fixed-effect model. And that in the comparison of 4–6 weeks and >6 weeks groups, the *I*^2 ^= 49% and the *P* for heterogeneity = 0.10. In the comparison of <4 weeks and >6 weeks groups, the *I*^2 ^= 50% and the *P* for heterogeneity = 0.09 (Figure 2). Because of that, fixed-effect model was fitting to be used. The OR in each comparision are 1.48 (95% CI, 0.98–2.25, *P* = 0.07), 0.84 (95% CI, 0.56–1.24, *P* = 0.38) and 1.07(95% CI, 0.66–1.75, *P* = 0.78), respectively (Figure 2). There are no publication bias found in pCR and the funnel plots were nearly symmetrical.

### R0 resection rate

**Figure 3:**
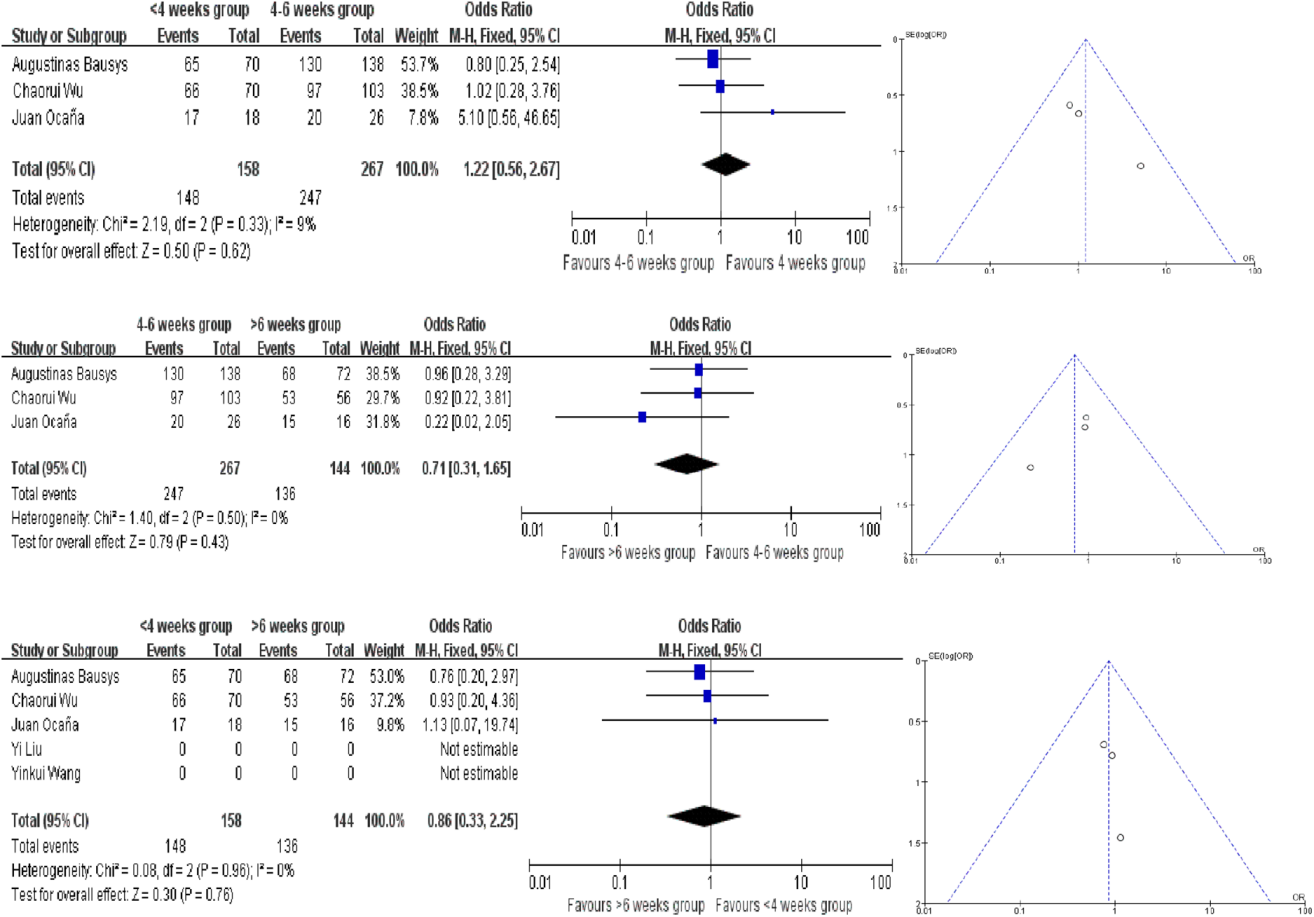
Forest plot and funnel plot for R0 resection rates meta analysis.

The R0 resection rates were reported in 3 studies, and no statistically significant difference was observed between the three different interval time groups. The ORs in each comparision are 1.22 (95% CI, 0.56–2.67, *P* = 0.62), 0.71 (95% CI, 0.31–1.65, *P* = 0.43) and 1.86 (95% CI, 0.33–2.25, *P* = 0.76). And all the analysis used the fixed-effect model according to the results of heterogeneity (Figure 3). In terms of R0 resection rates, no evidence of publication bias were found.

### Severe postoperative mortality

**Figure 4:**
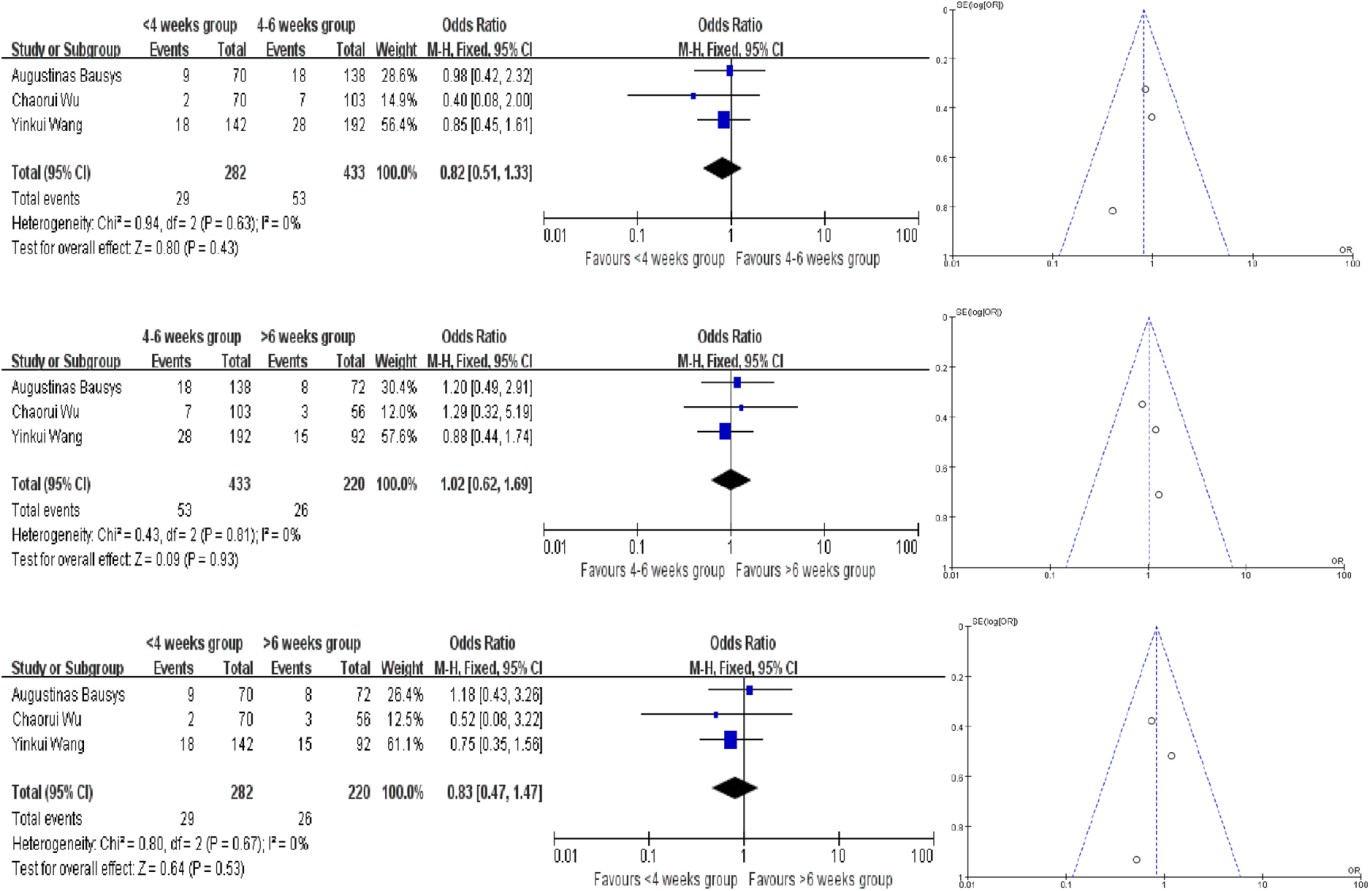
Forest plot and funnel plot for severe postoperative mortality rates meta analysis.

In this meta -analysis, only 3 studies provided the severe postoperative mortality rates, and we have not find the evidence to proof which interval time has the lower severe postoperative mortality rates. We also used the fixed-effect model to analyze and the ORs in each comparision are 0.82 (95% CI, 0.51–1.33, *P* = 0.43), 1.02 (95% CI, 0.62–1.69, *P* = 0.93) and 0.83 (95% CI, 0.47–1.47, *P* = 0.53) (Figure 4).

### 3-year PFS and OS rates

**Figure 5:**
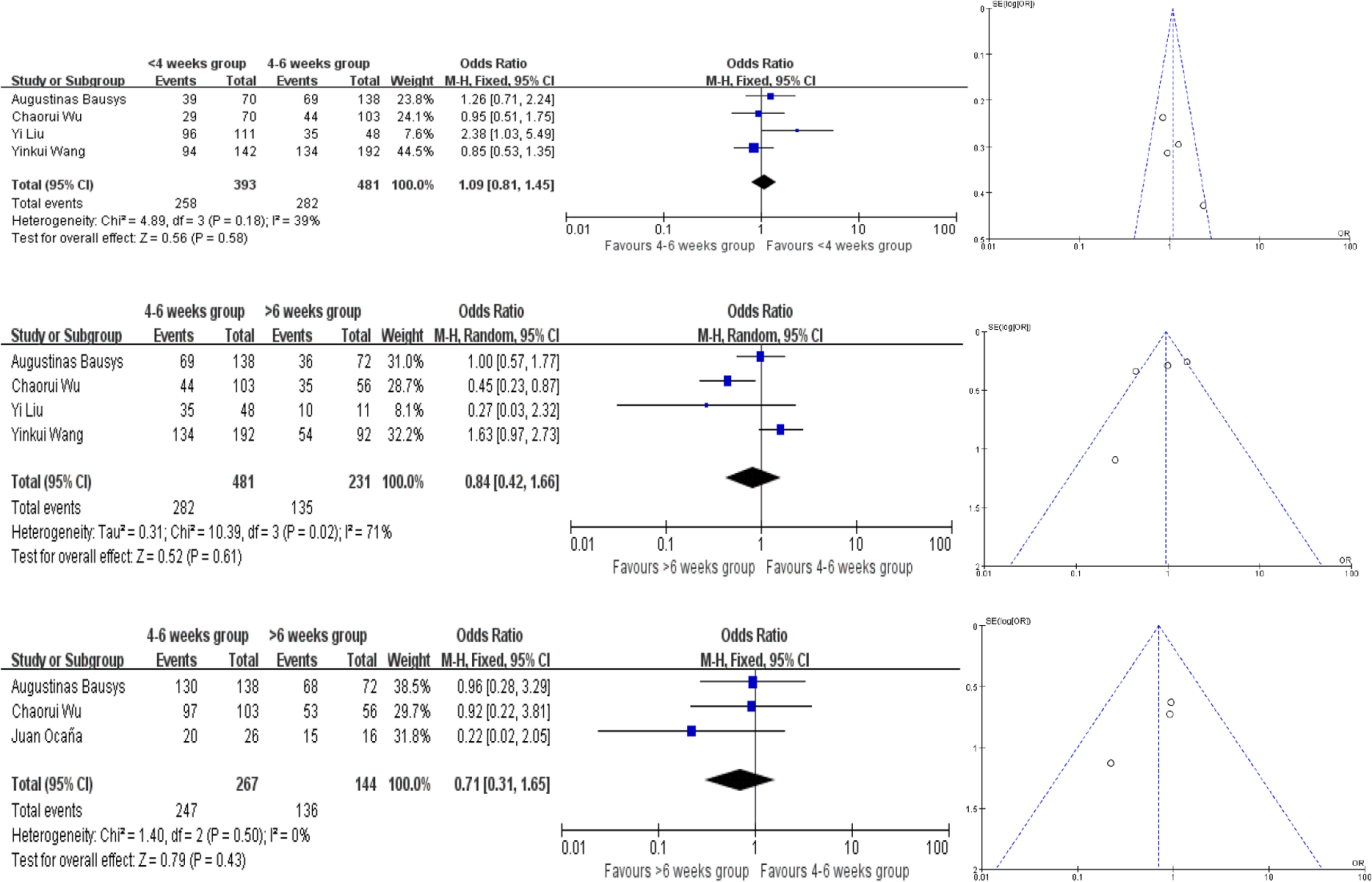
Forest plot and funnel plot for 3-year PFS rates meta analysis.

**Figure 6:**
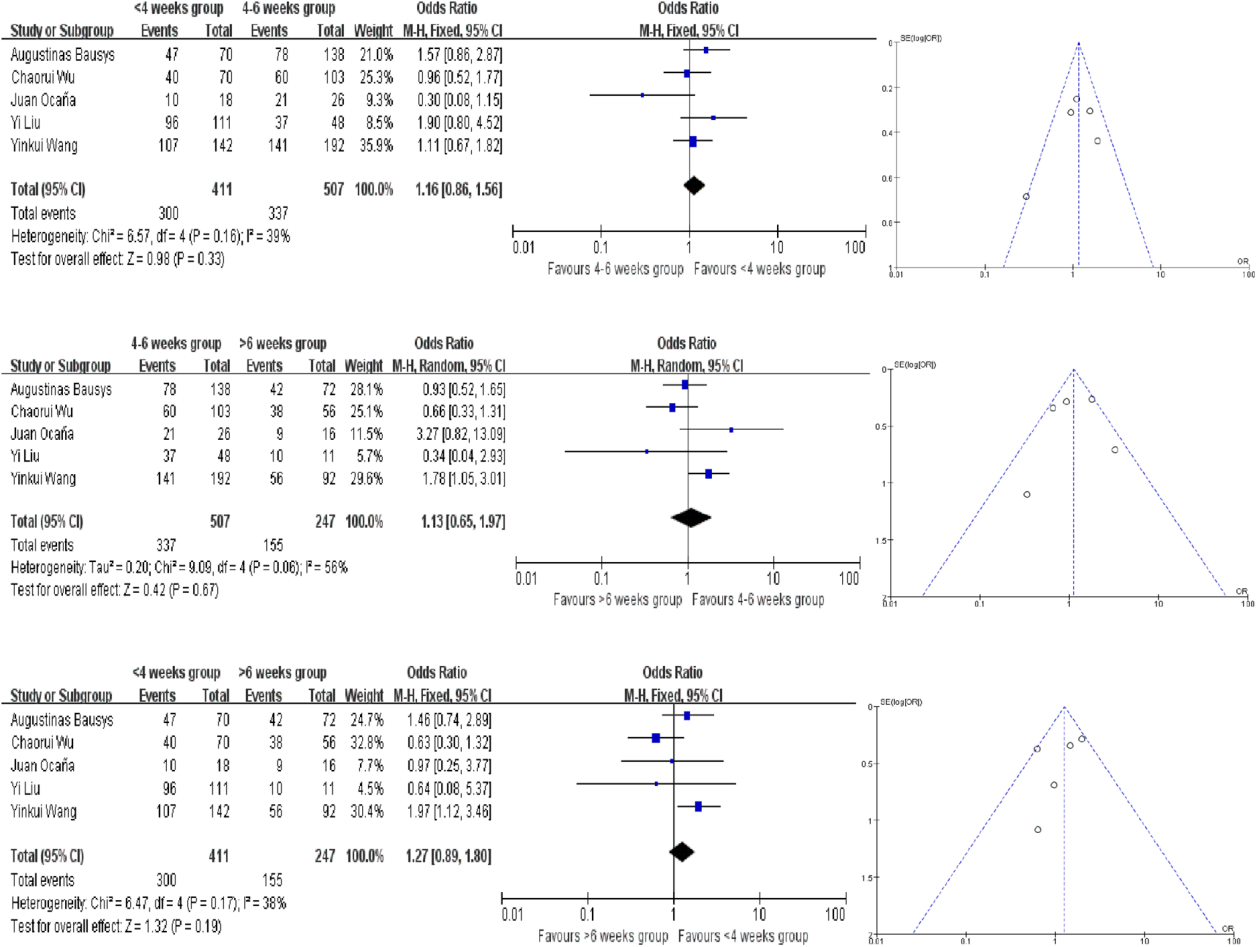
Forest plot and funnel plot for 3-year OS rates meta analysis.

Only 1 study provided the survival rate, but all of these studies provided the Kaplan–Meier survival curves. We extracted the survival data by using Engauge Digitizer 4.1. And there are no statistically significant difference in the different groups. And all the analysis used the fixed-effect model except for the comparison of 4–6 weeks group and >6 weeks group in terms of PFS and OS (Figures 5, 6). The PFS ORs in each comparision are 1.09 (95% CI, 0.81–1.45, *P* = 0.58), 0.84 (95% CI, 0.42–1.66, *P* = 0.61) and 0.71 (95% CI, 0.31–1.65, *P* = 0.43). And the OS ORs are 1.16 (95% CI, 0.86–1.56, *P* = 0.33), 1.13 (95% CI, 0.65–1.97, *P* = 0.67) and 1.27 (95% CI, 0.89–1.80, *P* = 0.19). Similar to the above, the funnel plots were nearly symmetrical.

## Discussion

In the current clinical work, there is still no definite conclusion on how long to perform surgery after chemotherapy in neoadjuvant chemotherapy for advanced gastric cancer. Most treatment protocols and large scale studies still chose the 4–6 weeks TTS as the standard interval time for neoadjuvant chemotherapy and surgery, but this “standard” is still not supported by large sample studies, and too small interval time may also lead to physical intolerance of GC patients (14, 15). So we carried out this meta analysis to clarify that. However, according to this present meta study, no differences were observed between the three groups divided by the different interval times in terms of the pCR rates, R0 resection rates, 3-year survival rates and severe postoperative complication rates. With the available research findings, we can infer that different interval times did not affect the efficacy of neoadjuvant chemotherapy in advanced gastric cancer. However, we also found that statistical differences between <4 weeks group and 4–6 weeks groups in terms of pCR rates were close to being present, suggesting that differences did exist between the two groups, but may have failed to manifest that due to small sample size of studies.

The design of gastrointestinal surgery, especially the timing of surgery after neoadjuvant chemotherapy, has been a difficult issue in relevant research. For the choice when to get the operation after the neoadjuvant chemotherapy, the following aspects are generally considered. Firstly, whether a shorter interval time will affect the patient's recovery after surgery, including the impact on the patient's quality of life, the incidence of postoperative complications and even more the survival time. Secondly, whether a longer interval time will lead to the tumour progression and the effect of neoadjuvant chemotherapy will be affected, failing to achieve the expected effectiveness of treatment such as the tumor radical resection and leading to tumour recurrence finally. Thirdly, tumour area were reduced after neoadjuvant chemotherapy but there were significant oedema of the tissue happening around the target lesion. Whether the oedema will normalise after a longer interval time and whether it will affect the patient's recovery and difficulty of surgery (16). In conclusion, choosing the right surgery timing for neoadjuvant chemotherapy patients may result in better survival benefits, as reflected in bigger possibility of tumour radical resection, less difficult to operate and longer term survival time after surgery.

In advanced gastric cancer, there are several studies involved the TTS. And the study by Yi Liu et al. found that incresing the interval time between the chemotherapy and surgery, especially to >6 weeks, may increase the pCR rate of GC patients, but the improvement in pCR did not benefit for GC patient survival time (9). In the authors' opinion, a more detailed interval differentiation for the >6 weeks group, such as adding a group of 8–12 weeks, might have gotten more accurate influence toward neoadjuvant chemotherapy. Meanwhile the smaller sample size calls into question the reliability of this study, so the follow-up studies are gradually being conducted. yinkui Wang improved the method of grouping by conducting a 21–84 day interval time study. A multiple group analysis was performed, and a interval time of 22–35 days, or 3–5 weeks, may have resulted in better survival benefits and did not reduce pCR rates or R0 resection rates (13). However, too close an interval (up to 21 days) may increase the risk of the incidence of postoperative complications in patients undergoing surgery, which may be caused by the alteration of the tumour microenvironment due to neoadjuvant chemotherapy (17, 18). At the same time, the effects of chemotherapy drugs can increase the degree of oedema in the surrounding tissues, making surgery more difficult and risky (19). However, Augustinas Bausys' study found the opposite, that earlier surgery may result in better surgical outcomes for patients (11). In general, there is still a lack of sufficient researches and there is no evidence from large samples studies to provide guidance on the most appropriate interval time.

In other solid tumours, particularly in the gastrointestinal tract, the determination of the interval time also remains controversial. A number of studies in esophageal cancer have found that longer intervals may improve pCR rates and R0 resection rates, with uncertain survival benefit, but this has been contradicted by the other studies. A meta-analysis by G. Lin in 2015 did not find a significant benefit in pathological response rates with longer intervals, but rather may be a risk factor for lower R0 resection rates (20). Also, prolonging the interval time (>7 weeks) reduced the 2-year overall survival rates of patients. However, a meta analysis by Qin Qin et al. in 2018, after further analysis of recent articles, found that longer intervals significantly increased the pCR rates of tumours, but also increased the rates of postoperative complications and reduced the overall survival rates of patients at 2 and 5 years (21). However, longer intervals did not change the R0 resection rate or the incidence of anastomosis-related complications. Therefore it is also extremely important to explore the suitable appropriate interval time in advanced gastric cancer.

We found, after the full search, that the interval time in gastric cancer was not found to affect the tumour response through the current study, nor was it found to be a risk factor for R0 resection and postoperative complications. This is not the same impression as that in previous studies and we can infer that the results of the current study do not be concluded that delaying or advancing the surgery after chemotherapy will result in a better survival benefit for patients (22). Therefore, in clinical work, it is sufficient to fully consider the patient's physical condition and perform surgery after assessing the surgical risk. The existence of so-called surgical time points has not been identified, and an appropriate extension of the interval between surgeries does not increase the risk of tumour progression for patients.

This study is the first meta-analysis to address the effect of interval time between neoadjuvant chemotherapy and surgery for gastric cancer. Five studies were included and data relating to 1,171 patients were analysed after a thorough literature search and extracting. The earliest study, by Yi Liu et al., was limited by a small sample size and unclear subgroups, which may have yielded conclusions of limited reliability (9). yinkui Wang performed detailed subgroups to obtain more precise time interval effects on patients (12). Both studies showed that an appropriate prolongation of TTS could lead to a survival benefit or a tumour regression benefit, but unlike the first two studies, Augustinas Bausys' study, which included 220 patients, obtained unfavourable results for prolonged TTS (11). This is the reason for conducting the present meta analysis. We hope that this first meta analysis can clarify whether the interval between neoadjuvant chemotherapy for gastric cancer affects patient prognosis. At present, there appears to be no evidence that TTS can affect the prognosis of neoadjuvant chemotherapy for patients with advanced gastric cancer.

Our study also had some limitations. The studies we included were retrospective and the randomisation of the studies was not controlled by the researchers. Also, not all of the five studies selected had items to be studied. The small number of studies is another shortcoming of this study. In addition, the criteria for mPR as proposed by Augustinas Bausys could be considered as a kind of regression grading criteria and is something that we could not determine more accurately. We also did not get the all 3-year OS and PFS data in these studies. The survival data may not match the source data because that we extracted the survival data by using Engauge Digitizer 4.1. But we thought we its accuracy is guaranteed and that a small amount of variation will not affect the results of the analysis. Finally, for the cutline in the study is beyond our control, some of the studies had different cut-off points and we had to choose a broader range. Therefore, more subsequent studies may be needed to flesh out our analysis.

## Conclusions

This study, based on an integrated analysis of previous studies on TTS in neoadjuvant chemotherapy for gastric cancer, did not find that TTS was associated with efficacy in neoadjuvant chemotherapy for advanced gastric cancer, nor did it find that prolonging or shortening TTS affected the safety of patients with gastric cancer. In clinical work, it is sufficient to take full account of the patient's physical condition and assess the risks of surgery before proceeding in a timely manner. Subsequent studies may provide assistance in developing relevant standards.

## Data Availability

The original contributions presented in the study are included in the article/Supplementary Material, further inquiries can be directed to the corresponding author/s.
